# Identification and functional activity of matrix-remodeling associated 5 (MXRA5) in benign hyperplastic prostate

**DOI:** 10.18632/aging.103175

**Published:** 2020-05-11

**Authors:** He Xiao, Ye Jiang, Weixiang He, Deqiang Xu, Ping Chen, Daoquan Liu, Jianmin Liu, Xinghuan Wang, Michael E. DiSanto, Xinhua Zhang

**Affiliations:** 1 Department of Urology, Zhongnan Hospital of Wuhan University, Wuhan, China; 2 Department of Urology, People’s Hospital of Qichun County, Huanggang, China; 3 Department of Surgery and Biomedical Sciences, Cooper Medical School of Rowan University, Camden, NJ 08103, USA; 4 Current address: Urological Surgery, Wuhan Children’s Hospital (Wuhan Maternal and Child Healthcare Hospital), Tongji Medical College, Huazhong University of Science & Technology, Wuhan, China

**Keywords:** benign prostatic hyperplasia, MXRA5, expression profiling by array, MAPK signaling pathway

## Abstract

Objective: Benign prostatic hyperplasia (BPH) is a common condition in aging males. The current study aims to identify differentially expressed genes (DEGs) associated with BPH and to elucidate the role of matrix-remodeling associated 5 (MXRA5) protein and mitogen-activated protein kinase (MAPK) signaling pathways in BPH.

Results: A total of 198 DEGs and a number of related pathways were identified with MXRA5 being one of the most significantly altered DEGs. MXRA5 was upregulated in BPH samples and localized mostly in stroma. Knockdown of MXRA5 induced stromal cell cycle arrest instead of inhibiting apoptosis. Consistently, MXRA5 overexpression enhanced epithelial cell proliferation. In addition, phosphorylated ERK1/2 and p38, key members of the MAPK family, were strongly decreased with knockdown but increased with overexpression.

Conclusion: Our novel data demonstrates that upregulation of MXRA5 in the enlarged prostate could contribute to the development of BPH through increasing cell proliferation via the MAPK pathway. Thus, the MXRA5-MAPK system could be rediscovered as a new therapeutic target for treating BPH.

Methods: Microarray analysis and integrated bioinformatics were conducted. The expression and biologic functions of MXRA5 was investigated via RT-PCR, western-blot, immunofluorescence, flow cytometry and MTT assay. Finally, genes involved in regulation of the MAPK pathway were investigated.

## INTRODUCTION

Benign prostatic hyperplasia (BPH) is one of the most common pathological diseases states in aging males [1], affecting approximately 50% of men in their fifties and 80% for men in their eighties. Lower urinary tract symptoms (LUTS) secondary to BPH greatly impact male quality of life [2, 3]. Despite the high prevalence, the pathogenesis of BPH still remains unclear. In recent decades, many hypotheses have been proposed including altered androgen-estrogen ratio, stromal-epithelial interactions, growth factors (Gfs) and cytokines, inflammation and autoimmune system, stem cells, and metabolic syndrome including obesity and diabetes, to likely play important roles in the onset and progression of BPH [4, 5]. Additionally, genetics, including a number of genes, contribute to the pathogenesis of this disease state. Indeed, there exists a familial form of BPH. Preliminary studies demonstrate DNA mutations [6], DNA hypomethylation [7], and abnormalities of nuclear matrix protein expression [8], along with miscellaneous genetic polymorphisms [9], and abnormal expression of the Wilms tumor gene (WT1) [10] in human BPH. Consequently, detailed analysis of the relevant pivotal genes is extremely beneficial. The establishment of the cDNA microarray technique provides the possibility to evaluate a huge quantity of genes. Indeed. Luo, et al. [11] have used 9 BPH specimens from men with extensive hyperplasia and 12 histologically normal prostates from radical prostatectomy specimens. The differentially expressed genes (DEG) from their microarray analysis were classified into four groups: (1) growth factors and their binding proteins, (2) hydrolases, proteases, and protease inhibitors, (3) stress response enzymes and (4) ECM molecules. Middleton, et al. [12] also used histologically normal prostate samples from patients undergoing radical prostatectomy as controls and demonstrated the stromal signaling molecules bone morphogenetic protein 5 (BMP5) and CXC chemokine ligand 13 (CXCL13) are enriched in BPH specimens while estrogen regulated pathways were depleted. In addition, they also performed single-cell RNA-Seq to verify their findings and whole-exome sequencing to uncovered somatic single-nucleotide variants in BPH. Different from the above two studies, Prakash, et al. [13] used prostate tissue from young donors as normal prostate, which also identified a genetic signature separating BPH from normal prostate. In our current study, we conducted a microarray analysis of five human BPH tissues and three normal prostate tissues and bioinformatics analysis of the DEGs was performed. Subsequently, we focused on the biologic functions of matrix-remodeling associated 5 (MXRA5) protein which is one of the most significantly altered DEGs.

Recent evidence suggests that BPH development involves accumulation of mesenchymal-like cells derived from the prostatic epithelium via epithelial-mesenchymal transition (EMT) [14–16]. The extracellular matrix (ECM) is a collection of extracellular molecules secreted by support cells that provides structural and biochemical support to the surrounding cells [17]. It not only serves as an external environment for the physiological activities of cells, but also supports the intercellular signal transduction pathways involved in multiple pathological and physiological processes. Moreover, androgens, estrogens and the ECM are demonstrated to have interactive effects [18]. Androgen and estrogen receptors regulate active peptides in tissues and stimulate cells to produce different components of the ECM. The ECM also interacts with hormones and growth factors to increase the sensitivity of prostate cells to androgen growth factors [19].

MXRA5 is a protein found in humans that is encoded by the MXRA5 gene. This protein contains 7 leucine-rich repeats and 12 immunoglobulin-like C2-type domains related to perlecan with a molecular weight of 312 kDa and is expressed in primates but not in rat or mouse, playing important roles in cell adhesion and matrix remodeling [20]. MXRA5 mRNA is up-regulated in human chronic ischemic myocardium and in fibroblast primary cultures from centenarians [21]. In addition to ECM remodeling, MXRA5 has been found to be involved in tumorigenesis. Somatic mutations of MXRA5 are observed in patients with non-small cell lung cancer (NSCLC) [22], MXRA5 is also aberrantly expressed in colorectal cancer (CRC) tissues, serving as a critical biomarker for the diagnosis and prevention of CRC [23]. The expression of MXRA5 in hyperplastic prostate has never been studied. However, MXRA5 has been recently found to be closely associated with the mitogen-activated protein kinases (MAPKs) pathways. It was demonstrated that the MAPK pathway plays a key role in the regulation of cell proliferation, survival, differentiation and apoptosis and is involved in signal transduction from the cell membrane to nucleus in response to a wide range of stimuli. Indeed, BPH could be a result of the imbalance between cell proliferation and cell apoptosis. Therefore, we postulate that MXRA5 is an important ECM factor involved in the remodelling of prostate tissue. In our current study, we conducted a microarray analysis comparing human BPH tissues to normal prostate tissues. Subsequently, we investigated the expression of both MXRA5 mRNA and protein in human hyperplastic prostate tissues. We further silenced and overexpressed the MXRA5 gene in human prostatic cells to identify those genes involved in the MAPK pathway.

## RESULTS

**Figure 1:**
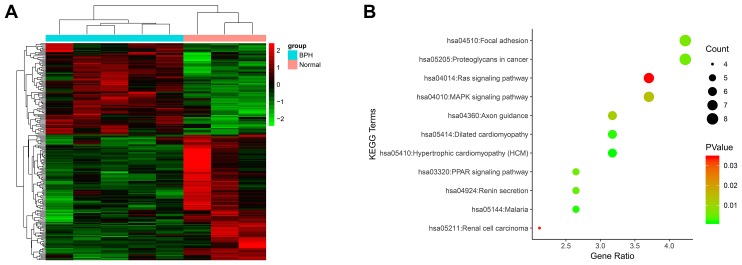
**Microarray analysis using mRNA isolated from BPH tissues and normal tissues.** (**A**) The heatmap plot of all 198 DEGs. The legend color bar on the right side indicates the relation between scaled expression values and colors, and the colors were balanced to ensure the black color represented zero value from comparison of five benign prostatic hyperplasia samples versus three normal prostate samples. (**B**) KEGG pathway analysis of 198 DEGs. The x-axis shows the number of genes and the y-axis shows the pathway terms. The negative log10 *P* value of each term is colored according to the legend. The count is indicated by the size of the circle.

To explore the biological mechanisms of BPH, we performed mRNA expression profiling of three normal prostates and five BPH tissues. A total of 198 DEGs were identified between the two groups (Figure 1A). This microarray data has already been submitted to the GEO database (serial number: GSE119195) and is the first microarray data on BPH that has been submitted to this database. MXRA5 is one of the highly altered DEGs and was of interest to us for further study. Based on the KEGG database, DEGs related pathways were also analyzed. As shown in Figure 1B, a number of pathways were found to be upregulated, including cell adhesion, Wnt signaling, and the Rap1 signaling, with the MAPK signaling pathway being most significantly altered (p < 0.01).

**Figure 2:**
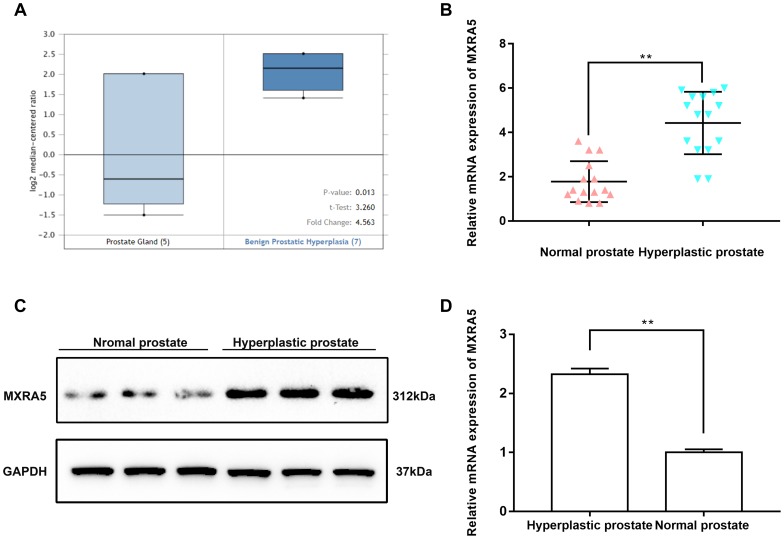
**MXRA5 is strongly upregulated in BPH tissues compared with the normal ones.** (**A**) Upregulation of MXRA5 mRNA expression in BPH analyzed by Oncomine database. Analysis using the Oncomine database revealed increased MXRA5 at transcriptional level in BPH stromal tissues versus normal prostate stroma. (**B**) qRT-PCR analysis showed that the gene expression of MXRA5 in BPH tissues (n = 15) was significantly higher than the normal prostate tissues (n = 15). The GAPDH mRNA was used as an internal control, ** means *P*<0.01. (**C**) Western blot analysis revealed a strongly increasing protein abundance of MXRA5 in BPH tissues versus normal ones. (**D**) Relative densitometric quantification of MXRA5 protein in BPH tissues versus normal ones. GAPDH expression was analyzed as a loading control, results are expressed as ratio of the proteins in respect to GAPDH. ** means *P* < 0.01 vs. normal prostates.

MXRA5 expression was further demonstrated in the Oncomine database with mRNA levels in BPH stroma being increased by 4.5 folds compared to controls (p = 0.013) (Figure 2A). For BPH and normal samples (n = 15) harvested at our institute, MXRA5 was found consistently upregulated over 2-fold both at the transcriptional and translational level (p < 0.01) (Figure 2B–2D)

**Figure 3:**
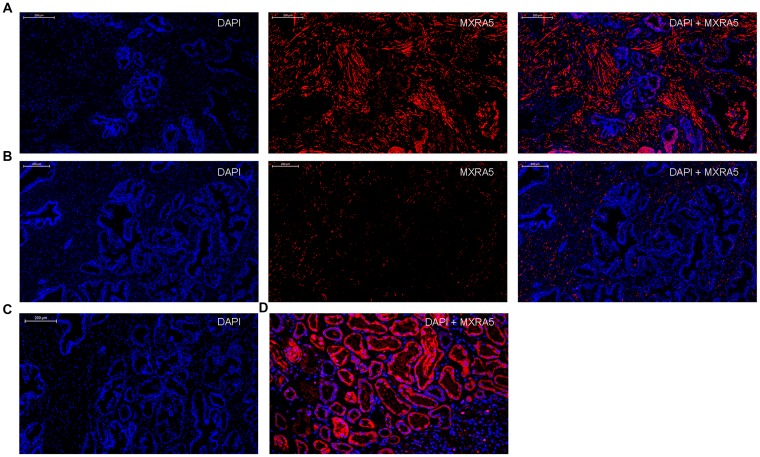
**Immunofluorescence localization of MXRA5 in human prostate tissues.** (**A**) Human BPH tissues. Left: DAPI (blue) indicates nuclear staining. Middle: Cy3-immunofluorescence (red) indicates the MXRA5 protein which was observed mainly in the fibromuscular stroma. Right: Merged image. (magnification ×200). (**B**) Human normal prostate. Left: DAPI (blue) indicates nuclear staining. Middle: Cy3-immunofluorescence (red) indicates MXRA5 protein. Right: Merged image (magnification ×200). (**C**) Negative controls omitting the primary antibody failed to stain. (**D**) Positive control using human renal cortex tissue showed a strong immune positivity for MXRA5 protein. DAPI (blue) indicates nuclear staining and Cy3-immunofluorescence (red) indicates MXRA5 protein staining (magnification ×200). Sections of all sample were used for immunofluorescence experiments and representative graphs were selected into figure.

**Figure 4:**
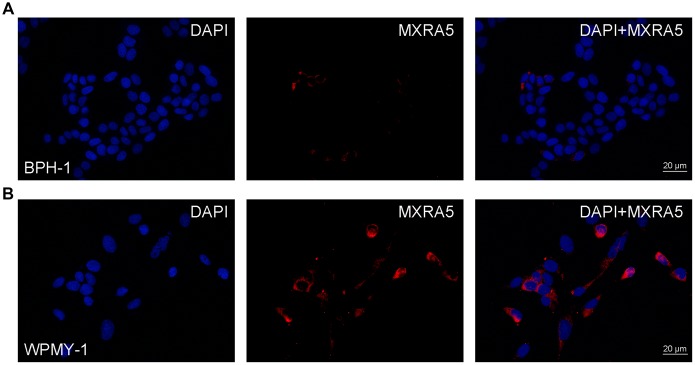
**Immunofluorescence of MXRA5 in human prostate cells.** (**A**) Human epithelial cells (BPH-1). Left: DAPI (blue) indicates nuclear staining. Middle: Cy3-immunofluorescence (red) indicates the MXRA5 protein which was rarely observed in the epithelial cells. Right: Merged image. The scale bar is 20 μm. (**B**) Human stromal cells (WPMY-1). Left: DAPI (blue) indicates nuclear staining. Middle: Cy3-immunofluorescence (red) indicates the MXRA5 protein which was abundantly observed in the stromal cells. Right: Merged image. The scale bar is 20 μm. Representative graphs of prostate cells were selected into figure.

Additionally, immunofluorescence staining demonstrated MXRA5 was predominantly localized in the stromal compartment of human prostate with lesser staining observed in the epithelium (Figure 3A). An overall increased MXRA5 staining was also observed in the enlarged prostate when compared with normal prostates using immunofluorescence microscopy (Figure 3B). Negative controls omitting the primary antibody failed to stain (Figure 3C) and positive controls using human renal cortex tissue showed a strong immune positivity (Figure 3D). Similarly, immunohistology demonstrated MXRA5 was present predominantly in stromal cells (Figure 4A) but to a much lesser degree in epithelial cells (Figure 4B).

**Figure 5:**
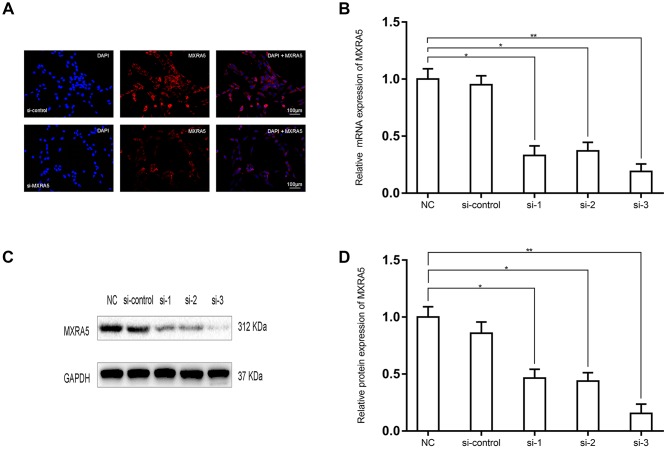
**Prostate cell lines exhibiting downregulated MXRA5 protein.** (**A**) Representative immunofluorescence staining of MXRA5 (red) in the WPMY-1 cells after si-MXRA5-3 treatment, compared with si-control treatment. Nuclei were stained by DAPI (blue). Three repeats of experiments for were conducted and representative graphs were selected into figure. The scale bar is 100 μm. (**B**) qRT-PCR validated the efficiency of distinct si-RNAs to knockdown MXRA5 at the transcriptional level in the prostate cells WPMY-1. All values shown are mean ± SD of triplicate measurements and repeated three times with similar results, * means *P*<0.05 and ** means *P*<0.01. (**C**) Western blot analysis showed a decreased protein expression of MXRA5 by the si-MXRA5 treatments, comparing to si-control treatment. (**D**) Relative densitometric quantification of MXRA5 in WPMY-1 cells. GAPDH expression was analyzed as a loading control, results are expressed as ratio of the proteins in respect to GAPDH. * means *P*<0.05 and ** means *P*<0.01 vs. si-control.

**Figure 6:**
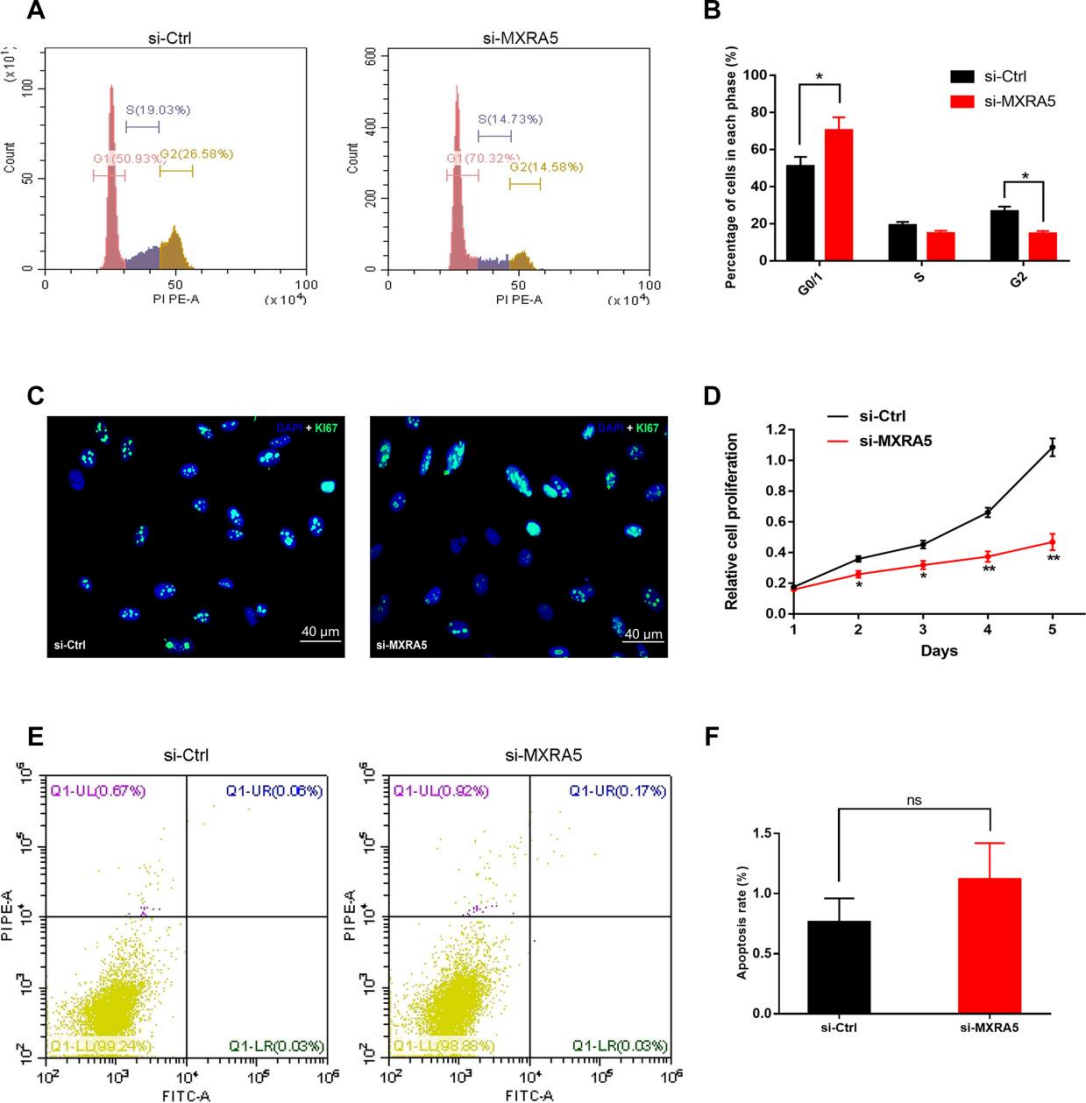
**Downregulation of MXRA5 represses prostate cell proliferation.** (**A**, **B**) Flow cytometry analysis for the WPMY-1 cells treated with si-MXRA5 for 48h compared with si-control treated cells. Percentages (%) of cell populations at different stages of cell cycles were listed within the panels. All histograms revealed the percentage (%) of cell populations from three independent experiments, * means *P* < 0.05. (**C**) Cell proliferation of WPMY-1 cells treated by si-control and si-MXRA5 was detected by Ki-67 staining (green). Nuclei were stained by DAPI (blue). Three repeats of experiments for were conducted and representative graphs were selected into figure. The scale bar is 40 μm. (**D**) MTT assay was used to detect the viability of the WPMY-1 cells treated by si-control (black line) and si-MXRA5 (red line). (**E**) Flow cytometry analysis of alterations of WPMY-1 cells apoptosis by the transfection using si-control and si-MXRA5. PI PE-A in y-axis stands for the fluorescence intensity of propidine iodide (PI) and FITC-A in x-axis stands for the fluorescence intensity of Fluorescein isothiocyanate (FITC) laballed Annexin V. Calculation area of the apoptosis rate was percentage of Annexin V+/PI+ cells. (**F**) Statistical analysis suggested no significant (ns) induction of apoptosis by the downregulation of MXRA5 in WPMY-1 cells. All values shown are mean ± SD of triplicate measurements and repeated three times with similar results, * means *P* < 0.05 and ** means *P* < 0.01.

**Figure 7:**
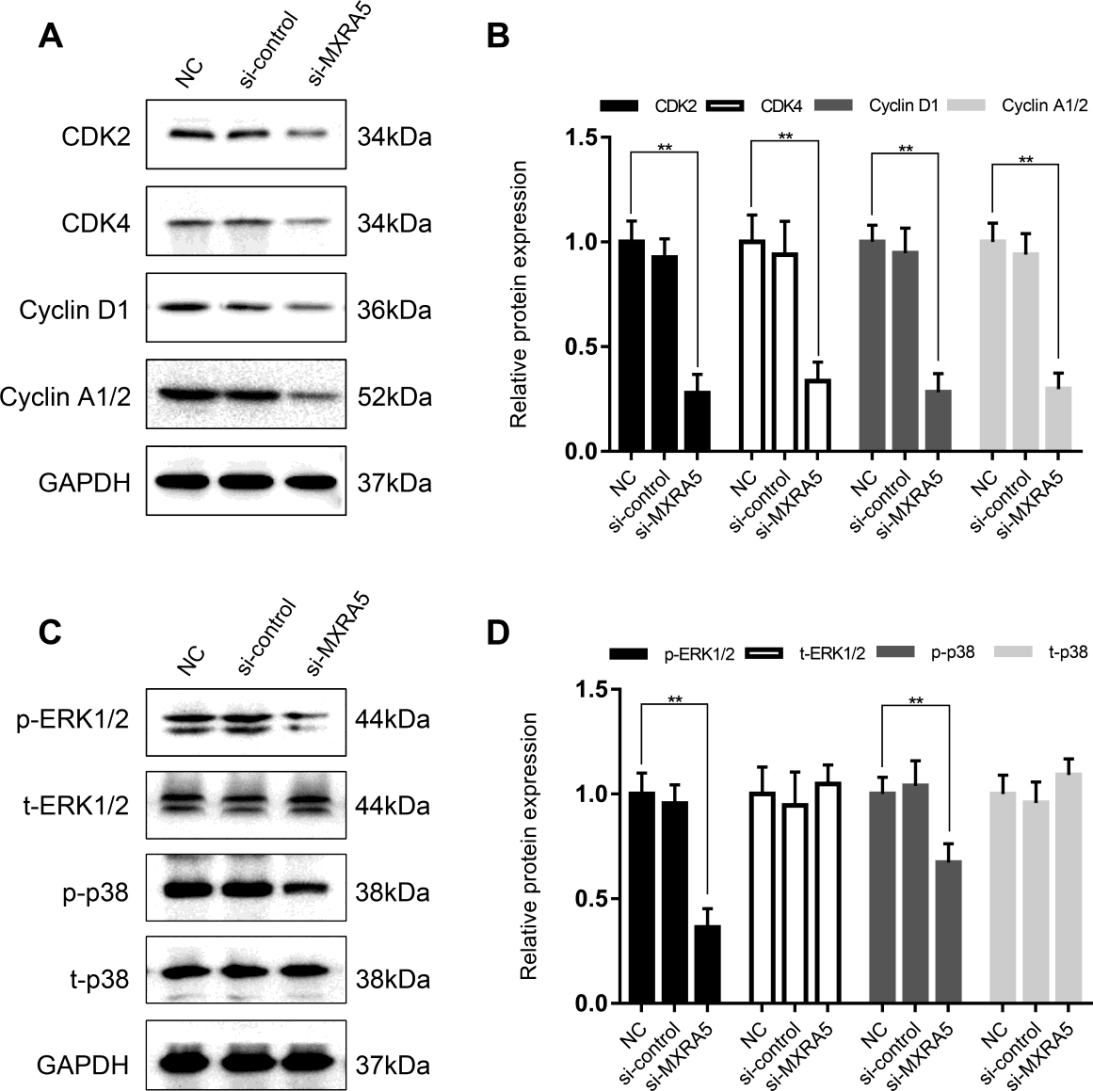
**MXRA5 participates in the regulation of cell cycle via MAPK pathway.** (**A**, **B**) Downregulation of proteins involved the cell cyle regulation (CDK2, CDK4, Cyclin D1 and Cyclin A1/2) in the WPMY-1 cells was revealed by Western blot analysis. GAPDH was used as a control and values of statistical data shown were mean ± SD of triplicate measurements and repeated three times with similar results. Statistical significance was calculated using ANOVA and ** means *P* < 0.01. (**C**, **D**) Western blot analysis for phosphorylated and total ERK1/2 as well as p38 MAPK (phosphorylated (p) and total (t). GAPDH was used as a control and ** means *P* < 0.01.

To create a cell model of MXRA5 deficiency, 3 distinct MXRA5-target-specific-siRNAs (si-MXRA5s) were transfected in to WPMY-1 cells. After 48 h, the knockdown efficiency was validated by qRT-PCR (Figure 5B) and Western blot analysis (Figure 5C, 5D). si-MXRA5-3 exhibited an inhibitory efficiency over 80% and was chosen for subsequent experimentation. Immunofluorescence staining showed the high expression of MXRA5 protein was strongly downregulated in si-MXRA5-3 transfected WPMY-1 cells (Figure 5A). Cell apoptosis and cell cycle stage were further analyzed for these transfected cells. A significant cell cycle arrest at the G0/G1 phase was determined with flow cytometry analysis (Figure 6A, 6B). Immunofluorescence staining showed that the MXRA5-siRNA group exhibited considerably less Ki-67 positive cells than the siRNA-control group (Figure 6C). Moreover, an MTT assay indicated that knockdown of MXRA5 restrained WPMY-1 proliferation drastically (Figure 6D). Additionally, expression proteins involved in G0/G1 phase regulation (cyclin A1/2, cyclin D1 and CDK2/4) was strongly decreased in MXRA5 silenced stromal cells (Figure 7A, 7B). However, no obvious effect on apoptosis was observed in si-MXRA5-3 transfected WPMY-1 cells (Figure 6E, 6F) as revealed by flow cytometry analysis. Finally, Western blot demonstrated that the protein levels of phospho-p38 MAPK (p-p38 MAPK) and p-ERK1/2 were significantly reduced with p38 MAPK and ERK1/2 unchanged following MXRA5-siRNA transfection (Figure 7C, 7D).

**Figure 8:**
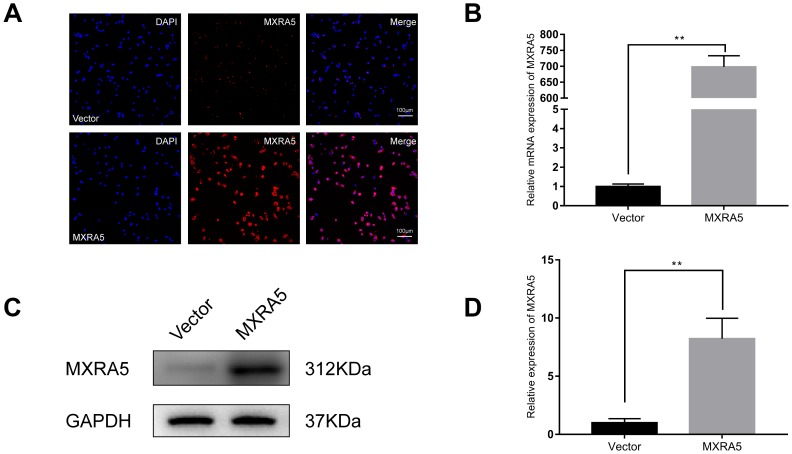
**Prostate cell lines with overexpression of MXRA5.** (**A**) Immunofluorescence staining of MXRA5 protein (red) in the BPH-1 cells after treatment with MXRA5-plasmid, compared with vector-control treatment. Nuclei were stained by DAPI (blue). Three repeats of experiments for were conducted and representative graphs were selected into figure. The scale bar is 100 μm. (**B**) qRT-PCR validated the efficiency of MXRA5 overexpression at transcriptional level in BPH-1 cells. All values shown are mean ± SD of triplicate measurements and repeated three times with similar results, ** means *P*<0.01. (**C**) Western blot analysis revealed an increased protein expression of MXRA5 by the MXRA5 plasmid treatment, compared to vector-control treatment. (**D**) Relative densitometric quantification of MXRA5 in BPH-1 cells. GAPDH expression was analyzed as a loading control, results are expressed as the ratio of the proteins with respect to GAPDH. ** means *P*<0.01 vs. vector-control.

**Figure 9:**
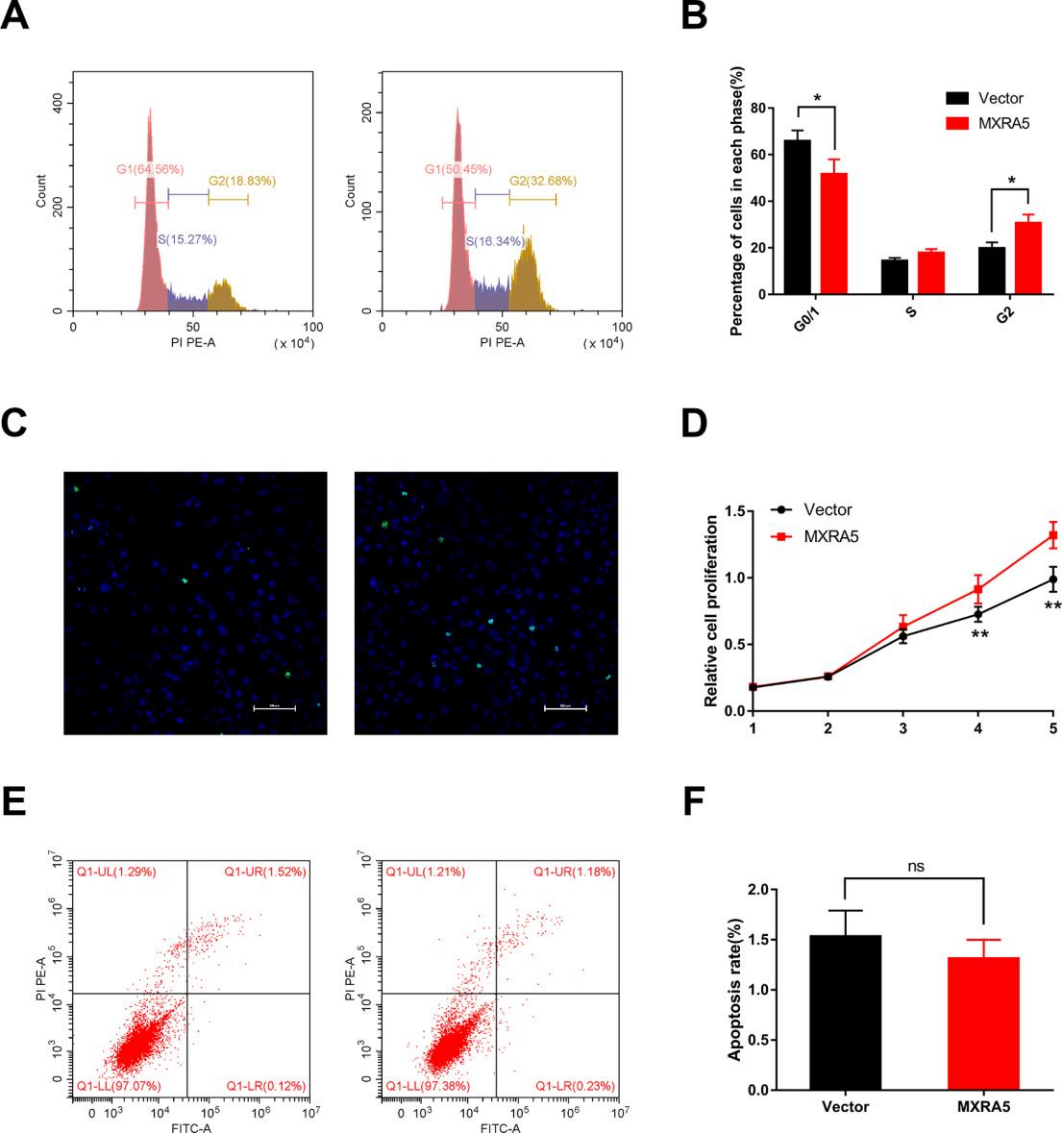
**Upregulation of MXRA5 promotes prostate cell proliferation.** (**A**, **B**) Flow cytometry analysis for BPH-1 cells treated with MXRA5 plasmid (Panel A: Right) for 48h compared with vector-control treated cells (Panel A: Left). Percentages (%) of cell populations at different stages of cell cycles were listed within the panels. All histograms revealed the percentage (%) of cell populations from three independent experiments, * means *P* < 0.05. (**C**) Cell proliferation of BPH-1 cells treated by vector-control (Left) and MXRA5 plasmid (Right) was detected by Ki-67 staining (green). Nuclei were stained by DAPI (blue). Three repeats of experiments for were conducted and representative graphs were selected into figure. The scale bar is 100 μm. (**D**) MTT assay was used to detect the viability of the BPH-1 cells treated by vector-control (black line) and MXRA5 plasmid (red line). (**E**) Flow cytometry analysis of alterations of BPH-1 cells apoptosis via transfection using vector-control (Left) and MXRA5 plasmid (Right). Calculation area of the apoptosis rate was percentage of Annexin V+/PI+ cells. (**F**) Statistical analysis suggested no significant (ns) induction of apoptosis by the upregulation of MXRA5 in BPH-1 cells. All values shown are mean ± SD of triplicate measurements and repeated three times with similar results.

**Figure 10:**
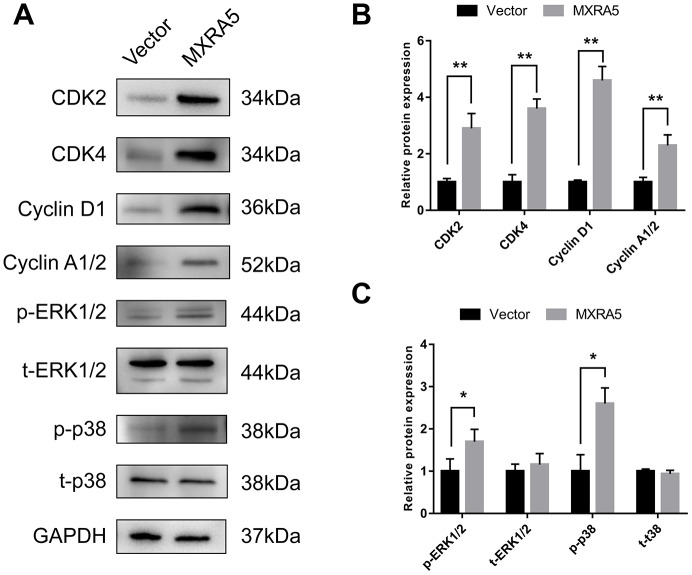
**MXRA5 participates in the regulation of epithelial cell cycle via MAPK pathway.** (**A**) Western blot of proteins involved in cell cyle regulation (CDK2, CDK4, Cyclin D1 and Cyclin A1/2) and phosphorylated (p) and total (t) ERK1/2 as well as phosphorylated (p) and total (t) p38 MAPK in the BPH-1 cells from Western blot analysis. GAPDH was used as a control. (**B**, **C**) Values of statistical data shown were mean ± SD of triplicate measurements and repeated three times with similar results. Statistical significance was calculated,* means *P* < 0.05 and ** means *P* < 0.01.

As MXRA5 is poorly expressed in prostate epithelial cells, plasmid was transfected into BPH-1 cells to over-express the MXRA5 protein. After 48h, efficiency of over-expression was determined by RT-PCR (Figure 8B) and Western blot analysis (Figure 8C, 8D) and MXRA5 was upregulated both at mRNA and protein levels. Immunofluorescence staining also showed the expression of MXRA5 protein was strongly upregulated in MXRA5-specific plasmid transfected BPH-1 cells (Figure 8A). Cell apoptosis and cycle were further analyzed for these transfected cells. In contrast to MXRA5 silenced stromal cells, the percentage of MXRA5 transfected BPH-1 cells at the G0/G1 phase was decreased while the percentage of cells at the G2 phase was increased (Figure 9A, Figure 9B). Immunofluorescence staining revealed that the MXRA5 group exhibited considerably more Ki-67 positive cells than the vector control group (Figure 9C). Moreover, MTT assay indicated that over-expression of MXRA5 significantly increased BPH-1 proliferation (Figure 9D). Additionally, proteins involved in G0/G1 phase regulation (cyclin A1/2, cyclin D1 and CDK2/4) were strongly upregulated in MXRA5 over-expression epithelial cells (Figure 10A, 10B). However, no obvious effect on apoptosis was also observed in MXRA5-specific plasmid transfected BPH-1 cells (Figure 9E, 9F) as revealed by flow cytometry analysis. Finally, Western blot demonstrated that the protein levels of phospho-p38 MAPK (p-p38 MAPK) and p-ERK1/2 were significantly increased with p38 MAPK and ERK1/2 unchanged following MXRA5-specific plasmid transfection (Figure 10A, 10C).

## DISCUSSION

In the current study, we performed a transcriptome analysis comparing BPH tissues versus normal prostatic samples. A total of 198 DEGs were identified with MXRA5 markedly upregulated. Along with these DEGs, a number of signaling pathways were altered in the prostate. Based on the Oncomine database, the MAPK pathway was the most significantly changed. The expression, localization and functional activity of MXRA5 were further elucidated using human hyperplastic prostate tissues and cultured human prostate cell lines. Our novel data demonstrated that the upregulation of MXRA5 in the enlarged prostate could contribute to the development of BPH via increasing cell proliferation instead of inhibiting cell apoptosis via phosphorylating ERK1/2 and p38 of the MAPK pathway.

BPH is a quite common condition in aging males with the incidence continuing to rise due to an increasing aging population worldwide. α 1-adrenoceptor antagonists [4] and 5α-reductase inhibitors [5] are efficacious nonsurgical treatments for BPH. However, around 30% of patients still require surgery. Moreover, the mechanisms of BPH are not completely understood. Thus, it is necessary to obtain a central knowledge of the molecular mechanisms involved in the etiology of BPH to discover new therapeutic strategies. As microarray analysis could precisely provide us differentially expressed genes and related biological functions, in this current study we conducted microarray analysis of 5 human prostatic hyperplasia tissues and 3 normal prostatic tissues from young donors. The microarray data were submitted to the GEO database and a serial number (GSE119195) was assigned. Until now, there has been no published microarray data available in the public database concerning benign prostate tissues.

Many of the prostatic stromal-epithelial interactions that are observed during normal development of prostate and BPH may be mediated by soluble growth factors or by the ECM, which itself has growth factor–like properties [24, 25]. Our microarray analysis identified hundreds of DEGs with MXRA5 being one of the most markedly changed, which is a matrix-remodeling related gene of interest to us. Indeed, MXRA5 mRNA levels in BPH stroma were increased by 4.5 fold when analyzing data from the Oncomine database. The increased expression of MXRA5 was further demonstrated via RT-PCR and Western blot assays using prostate tissues from our institute. Both mRNA and protein levels were significantly upregulated in human hyperplasia prostatic tissues when compared with normal ones. MXRA5 has been reported to be expressed in primates, marsupials, some mammals, birds and fish, but not in rat or mouse [26]. Indeed, we did not observe MXRA5 staining in rat prostate (data not shown). Our present study also demonstrated that MXRA5 was mainly localized in the stroma of human prostate [27].

As our understanding of stromal-epithelial cell relationships in the prostate increases, it is possible that therapies may be designed to induce regression of established BPH by modulating one class of stromal cell excretory protein (i.e., ECM, such as MXRA5). In turn, these proteins regulate epithelial cell differentiation. The present study created a MXRA5 deficiency cell model. As MXRA5 was mainly localized in stroma, cultured stromal cells (WPMY-1) were transfected with siRNA to knockdown MXRA5. The silencing of MXRA5 ameliorated WPMY-1 proliferation drastically and a significant cell cycle arrest at G0/G1 phase was observed. However, knockdown of MXRA5 had no obvious effect on apoptosis in WPMY-1 cells. We also co-cultured siMXRA5 transfected WPMY-1 cells with BPH-1 cells with no inhibitory effect observed on epithelial cells (data not shown). But when a MXRA5 overexpressed epithelial cell model was created, enhanced BPH-1 cell proliferation and accelerated cell cycle from G0/G1 phase to G2 phase were observed. MXRA5 has been reported to be a novel biomarker which is upregulated in non–small cell lung carcinoma [22, 28] and colorectal cancer [23]. Therefore, MXRA5 could modulate both prostate stromal cell and epithelial cell proliferation.

Our microarray analysis also suggested that the MAPK signaling pathway was linked with BPH. When the growth regulation and the homeostatic control between proliferative and non-proliferative behavior in the periurethral and transition zones of the prostate are disrupted, they lead to the expansion of the cell proliferating compartment and the concomitant decrease in the extent of the differentiated compartment [29]. Histologically, BPH represents hyperplasia of both the stromal and glandular components of the prostate, with stromal smooth muscle elements comprising approximately 40% of the cellular volume of the enlarged prostate [30]. This is the end result of an ensemble of different stimuli and local conditions that are influenced by androgen metabolism, stromal-epithelial interactions with autocrine–paracrine secretion of GFs and the action of cytokines produced by inflammatory cells [29, 31]. There is indirect evidence that all three MAPK cascades might be implicated in these pathological alterations by modulating the local prostatic environment to favor the progression of BPH. It is known that MAPK family members participate in regulating the cell cycle in various ways [32]. ERK mainly is involved in regulating the progression of G0/G1 to the S phase and p38 primarily regulates the G2 checkpoint [33, 34]. Interestingly, a recent study reported that the MXRA5 protein is a positive regulator of the MAPK signaling pathway in preeclampsia [20]. Similarly, the current study found that MXRA5 knockdown induced prostatic stromal cell cycle arrest at G0/G1 phase which was confirmed by downregulation of related proteins (cyclin D1 and CDK2/4) and MXRA5 over-expression significant decreased the percentage of epithelial cells at the G0/G1 phase. Consistently, our study also showed that p-p38 and p-ERK1/2 were significantly reduced with p38 MAPK and ERK1/2 unchanged following MXRA5 knockdown, p-p38 and p-ERK1/2 were significantly increased with p38 MAPK and ERK1/2 unchanged following MXRA5 overexpression, suggesting MAPK has important roles in regulating cell proliferation [35] via connecting extracellular stimuli from the cell membrane to the nucleus. Currently, the first-line therapies for BPH are based on the physiology of the prostate with α1-blockers (reduce the prostate tone) and 5α-reductase inhibitors (reduce the prostate volume). However, these treatments cannot completely prevent the progression of BPH. The present study identified genetically altered genes and related signaling pathways of BPH. Therefore, treatment based on pathophysiology of BPH could play a more important role for the future therapy for BPH during routine clinical practice.

There are several factors that may have influenced the outcome of the current study. First, the difference in age between groups may influence the outcome with normal prostate samples being obtained from young donors and BPH samples obtained from aging males. Thus, some differentially expressed genes (DEGs) identified in the present study may be attributed to different age, instead of BPH. However, Luo J, et al. [11] harvested the normal prostate samples from histologically normal regions within the radical prostatectomy specimens. They compared gene expression profile from normal samples with age ≥59 ys and BPH samples with age ≤59 ys and showed similar alterations as in their analysis with the presence of age difference (52.5 vs. 70.6, normal vs. BPH). Consistently, our current microarray analysis identified the same DEGs as theirs, such as BMP5, NELL2, IGF1. Thus, age should not have much influence on the outcomes of our study. Second, origin of tissue may also influence the outcome since different prostate zones have specific architectural and stromal feature. To avoid this interference, samples in our study were all collected from the prostate transition zone. Third, hyperplastic prostate might have cancer-related genetic changes since they were obtained from patients undergoing cystoprostatectomy for infiltrating bladder cancer. However, the prostate samples we used showed no tumor infiltration.

Collectively, this is the first study to reveal both MXRA5 gene and protein expression increased in hyperplastic prostate, while knockdown of MXRA5 inhibited and overexpression of MXRA5 enhanced prostatic stromal cell cycle. Moreover, this process may be partially connected with alteration of the MAPK signaling pathway. Our data suggests that the MXRA5-MAPK system could play important roles in the development of BPH and it could be rediscovered as new therapeutic targets for BPH. Nevertheless, there is some limitation in our study. It will be better to establish a BPH rat model to explore the expression and functional activities of MXRA5 in animal prostate. Unfortunately, rodent animals do not express MXRA5 protein. In the future, genetic mice with overexpressed MXRA5 gene could be an intriguing model to explore the in vivo function of MXRA5.

## MATERIALS AND METHODS

### Ethical statement for human prostate tissue samples

Fifteen samples from young brain-dead men undergoing organ donation were obtained as controls and fifteen BPH samples were obtained from patients undergoing cystoprostatectomy for infiltrating bladder cancer without prostate infiltration. The prostate transition zone was procured and utilized for all experiments. All human samples were obtained after the approval of the Hospital Committee for Investigation in Humans and after receiving written informed consent from all patients or their relatives involved. Prostate tissues were divided into 3 pieces and were respectively stored in RNA Sample Protector (Takara Bio. Inc., Otsu, Shiga, Japan) for PCR analysis, 10% neutral buffered formalin for histological examination, liquid nitrogen for Western blot analysis and immunofluorescence microscopy. All human studies were conducted in accordance with the principles of the Declaration of Helsinki. The study was approved by the Ethics Committee at Zhongnan Hospital of Wuhan University and the sample collection as well as treatment were carried out in accordance with the approved guidelines.

### Human prostatic cell lines

SV40 large T antigen-immortalized stromal cell line WPMY-1 (Cat. #GNHu36) was purchased from the Stem Cell Bank, Chinese Academy of Sciences in Shanghai, China. Human benign prostatic enlargement epithelia cell line BPH-1 (Cat. #BNCC339850) was purchased from the Procell Co., Ltd. in Wuhan, China. Identification of the cell lines was performed at the China Center for Type Culture Collection in Wuhan, China. WPMY-1 cells were cultured in DMEM medium (Gibco, China) containing 1% penicillin G sodium/streptomycin sulphate and 5% FBS and BPH-1 cells were cultured in RPMI-1640 medium (Gibco, China) containing 10% fetal bovine serum (FBS) (Gibco, Australia) in a humidified atmosphere consisting of 95% air and 5% CO_2_ at 37 ºC.

### Total RNA isolation from prostate tissues and cells

Total RNA was isolated from prostate tissues and cells using the Qiagen RNeasy Mini Kit (Cat #74101), combined with QIAshredder from Qiagen (Cat #79654) using a centrifuge (Eppendorf, Cat #5424) to increase the quantity and quality of isolated total RNA, according to the manufacturer’s protocol. Each RNA sample was digested by DNase I (RNase-Free DNase Set, Qiagen, Cat #79254) to remove possible contamination of genomic DNA. The quantity of isolated RNA was measured by a NanoDrop® ND-1000 UV-Vis spectrophotometer (Thermo Scientific, USA).

### Microarray analysis of mRNA isolated from human prostate tissues

Microarray analysis was performed according to the standard Affymetrix protocol [36]. Briefly, 250 ng total RNA isolated from each BPH tissue and the normal prostate tissues was prepared to biotinylated cDNA by Ambion® WT Expression Kit. On GeneChip Human Transcriptome Array 2.0, 5.5 μg of cDNA were hybridized for 16 h at 45ºC, continuously washed and stained in the Affymetrix Fluidics Station 450, scanned by using Affymetrix® GeneChip Command Console (AGCC) installed in GeneChip® Scanner 3000 (7G). Data analysis was with a Robust Multichip Analysis (RMA) algorithm using Affymetrix default analysis settings and global scaling as normalization method. Pathway analysis was performed on the Database for Annotation, Visualization, and Integrated Discovery (DAVID) [37] to analyze the main function of the DEGs. We used the 198 identified DEGs for the query, with the whole human genome as the background. The analysis was performed using the DAVID default parameters. Pathway analysis was performed on the basis of the Kyoto Encyclopedia of Genes and Genomes (KEGG) to identify the significantly enriched pathways of DEGs [38].

### Reverse transcription and quantitative real time PCR (qRT-PCR)

1 μg of total RNA isolated from prostate tissues or cells was mixed with oligo (dT) 12–18 primers to synthesize first-strand cDNA by using RevertAid First Strand cDNA Synthesis Kit (Thermo Scientific, China). 1 μg of cDNA was used for each polymerase chain reaction (PCR) in a final volume of 20 μl. All primers used with the SYBR Premix Ex Taq II (Takara Bio, China) were tested for optimal annealing temperatures and PCR conditions were optimized with gradient PCRs on a Bio-Rad iCycler (Cat. #CFX96). Primer sequences and annealing temperatures are summarized in Table 1. Values were normalized for amplified β-actin alleles. Relative gene abundance = 2^-ΔΔct^, Δct = ct_target gene_ - ct_gapdh_, for cells ΔΔct = Δct_siRNA(or plasmid)-treated_ - Δct_siRNA-untreated_, for prostate tissues ΔΔct = Δct_BPH tissues_ -Δct_normal prostate tissues_ (ct = threshold cycle).

**Table 1:** The primers for qRT-PCR.

**Gene**	**Symbol**	**Forward primer(5'-3')**	**Reverse primer(5'-3')**	**Annealing temperature (°C)**	**Length (bp)**
Matrix-remodeling associated 5	MXRA5	CCTTGTGCCTGCTACGTCC	TTGGTCAGTCCTGCAAATGAG	61	152
Glyceraldehyde-3-phosphate dehydrogenase	GAPDH	TGCACCACCAACTGCTTAG	GATGCAGGGATGATGTTC	60	176

### Knockdown of MXRA5 in the WPMY-1 cells

MXRA5-target specific small interfering RNA (siRNA) was synthesized by Genepharma Ltd. in Suzhou, China. WPMY-1 was transfected with MXRA5-siRNA (si-MXRA5) using lipofectamine 2000 (Invitrogen, USA), according to the manufacturer’s protocol. The sense sequences of si-MXRA5 are as follows: si-1, 5'-GCACCUGACAUUAAGAUUUTT-3'; si-2, 5'-CCACAAGUCCUCCAUCCUUTT-3'; si-3, 5'-GCUUCCAGAUACUUUGUAATT-3'. And the sense sequence of si-control is 5'-UUCUCCGAACGUGUCACGUTT-3'. After transfection by si-MXRA5 for 48 h, alterations of MXRA5 at transcriptional and protein levels were evaluated by the qRT-PCR, Western blot and immunofluorescence microscopy staining analyses.

### Over-expression of MXRA5 in BPH-1 cells

MXRA5 cDNA was polymerase chain reaction (PCR) amplified from a cDNA library of human prostate cell lines and then cloned into a 2 × FlagpcDNA3 empty vector performed with a one-step method to construct the homologous recombination vectors. After transfection by plasmid for 48 h, alterations of MXRA5 at transcriptional and protein levels were evaluated by the qRT-PCR, Western blot and immunofluorescence microscopy staining analyses.

### MTT assay

After transfection for 48 h, 3000-5000 WPMY-1 or BPH-1 cells/200 μl medium were seeded in 96-well plates to grow for another four days. Then 20 μl MTT was added in each well and incubated at 37°C for 4 h. After removing the medium, formazan precipitate was dissolved in DMSO, and absorbance at 490 nm was measured by a microplate reader (cat. no. SpectraMax M2; Molecular Devices, Sunnyvale, CA, USA).

### Flow cytometry analysis for alterations of cell cycle and apoptosis

For cell cycle analysis, 1 × 10^6^ cells were harvested and fixed in 70% ice cold ethanol at –20ºC overnight. After centrifugation, pellets were resuspended with PBS containing 50 μg/ml propidium iodide (Sigma-Aldrich, USA) and 0.1 mg/ml RNaseA (20 μg/ml in PBS) in the dark. After incubation at 37ºC for 30 min, the DNA content distribution was analyzed by flow cytometry analysis (Beckman, Cat. #FC500). Cell apoptosis was analyzed by flow cytometry analysis using Annexin V-fluorescence isothiocyanate (FITC)/PI apoptosis detection kit (BD Biosciences, San Jose, CA, USA), according to the manufacturer's instructions.

### Western blot analyses

Proteins were extracted from frozen samples using the Radio Immunoprecipitation Assay Lysis Buffer (Shanghai Beyotime Biotechnology Co., Ltd., Shanghai, China) and 100 μg of each sample was eletrophoresed on a 10% sodium dodecyl sulfate-polyacrylamide (SDS-PAGE) gel (Wuhan Boster Biological Technology Ltd., Wuhan, China) and transferred to polyvinylidene fluoride (PVDF) membrane (Millipore, Billerica, MA, USA) using a BioRad wet transfer system. The membrane was blocked for 2 h at room temperature with Tris-buffered saline with 0.1% [v/v] Tween (TBST) containing 5% [w/v] non-fat dry milk solution. The membrane was incubated overnight with primary MXRA5 antibody at dilution of 1:1000 (Abcam, Cambridge, UK). Membranes were washed with TBST three times and incubated at room temperature for 1 h with an IRDye 800CW conjugated goat anti-rabbit IgG (LI-COR, Lincoln, USA) at dilutions of 1:15000. After washing, the blots were visualized by scanning using a Li-Cor Odyssey Imager (Li-Cor, Lincoln, USA). The bands were quantified by densitometry using Li-Cor Odyssey software. A monoclonal rabbit antibody against GAPDH (1:10000; Abcam, ab181602) was used as a control to ascertain equivalent loading.

### Immunofluorescence staining for prostate cells

Coverslips containing cells were washed 3 times by cold PBS and fixed with 4% PFA for 30 min. Then the cells were treated with 0.1% Triton X-100 and blocked in goat serum for 30 min, incubated with primary antibody (Table 2) at room temperature for 2 h, washed with PBS and incubated with Cy3-labeled or FITC-labeled secondary antibody (Table 3) for 1 h. Nuclei were labeled with DAPI (2 μg/ml). Immunofluorescence staining was analyzed using a fluorescence microscope (cat. no. IX73; Olympus, Japan).

**Table 2:** List of primary antibodies.

**Antigens**	**Species antibodies raised in**	**Dilution (IF)**	**Dilution (WB)**	**Supplier**
CDK2, human	Rabbit, monoclonal		1:2000	Cell Signaling Technology, USA, Cat. #2546
CDK4, human	Rabbit, monoclonal		1:2000	Cell Signaling Technology, USA, Cat. #12790
Cyclin D1, human	Rabbit, monoclonal		1:2000	Cell Signaling Technology, USA, Cat. #2978
Cyclin A1/2, human	Rabbit, monoclonal		1:1000	Abcam, UK, cat. no.ab185619
Phospho-p44/42 MAPK (Erk1/2) (Thr202/Tyr204), human	Rabbit, monoclonal		1:1000	Cell Signaling Technology, USA, Cat. #4370
Glyceraldehyde 3-phosphate dehydrogenase (GAPDH),human	Mouse, monoclonal		1:2000	Santa Cruz Biotechnology Inc., USA, Cat. #sc-365062
Phospho-p38 (Thr180/Tyr182), human	Rabbit, monoclonal		1:1000	Cell Signaling Technology, USA, Cat. #4511
p38 MAPK, human	Rabbit, monoclonal		1:2000	Cell Signaling Technology, USA, Cat. #8690
p44/42 MAPK(Erk1/2), rat	Rabbit, monoclonal		1:2000	Cell Signaling Technology, USA, Cat. #4695
MXRA5	Rabbit, monoclonal	1:100	1:1000	Abcam, UK, cat. no.ab209075

**Table 3:** List of secondary antibodies and counterstaining of nuclei.

**Secondary detection system used**	**Host**	**Method**	**Dilution**	**Supplier**
Anti-Mouse-IgG (H+L)-HRP	Goat	WB	1:10,000	Sungene Biotech, China, Cat. #LK2003
Anti-Rabbit-IgG (H+L)-HRP	Goat	WB	1:10,000	Sungene Biotech, China, Cat. #LK2001
Anti-rabbit IgG (H+L), F(ab')2 fragment (Alexa Fluor® 488 Conjugate)	Goat	IF	1:50	Cell Signaling Technology, USA, cat. no. 4412
Hoechst 33342 (1 mg/ml) nucleic acid staining (DAPI)	-	IF	1:750	Molecular Probes/Invitrogen, Carlsbad, CA, USA, cat. no. A11007

### Immunofluorescence staining for BPH and normal samples

All the samples were fixed by 4% PFA at 4ºC overnight and embedded into paraffin (Paraplast, Sigma-Aldrich) using a tissue processor (Thermo Fisher Scientific, Cat. #STP 120). Paraffin sections (4 μm) were cut with a rotary microtome (Thermo Fisher Scientific, Cat. #HM325). The sections were serially incubated with indicated primary antibody (listed in Table 2) and Cy3-labeled or FITC-labeled secondary antibody (listed in Table 3) in a humidified atmosphere. Nuclei were labeled with DAPI (2 μg/ml). Sections were analyzed via a fluorescence microscope (Olympus, Cat. #IX73).

### Statistical analyses

Results are expressed as mean ± standard deviation (SD) in tables and mean ± standard error of mean (SEM) in bar graphs. Student’s t-test was performed by GraphPad Prism v7.0 software (two sample treatments compared) to analyze the differences between two groups. P < 0.05 was considered statistically significant.

### Ethical approval

All human samples were obtained after the approval of the Hospital Committee for Investigation in Humans and after receiving written informed consent from all patients or their relatives involved. All human study was conducted in accordance with the principles of the Declaration of Helsinki. The study was approved by the Ethics Committee at Zhongnan Hospital of Wuhan University and the sample collection as well as treatment were carried out in accordance with the approved guidelines.
